# Processing Optimization for Metal Injection Molding of Orthodontic Braces Considering Powder Concentration Distribution of Feedstock

**DOI:** 10.3390/polym12112635

**Published:** 2020-11-10

**Authors:** Chao-Ming Lin, Jhih-Jyun Wu, Chung-Ming Tan

**Affiliations:** 1 Department of Mechanical and Energy Engineering, National Chiayi University, Chiayi 60004, Taiwan; a5709ty56@gmail.com; 2 Department of Mechanical Engineering, WuFeng University, Chiayi 62153, Taiwan; cmtan@wfu.edu.tw

**Keywords:** metal injection molding, Taguchi method, orthodontic brace, powder concentration distribution, mold flow analysis

## Abstract

Metal injection molding (MIM) utilizes a compound consisting of metal powder particles and a binding agent as the feedstock material. The present study combines MIM mold flow simulations with the Taguchi method to clarify the individual and combined effects of the main MIM process parameters on the metal powder concentration distribution in the final sintered product. The results show that the molding process should be performed using a short filling time, a high melt temperature, a low packing pressure, a low mold temperature, and a small gate size. Given these process settings, the powder concentration uniformity and phase separation effect are significantly improved; giving rise to a better aesthetic appearance of the final sintered product and an enhanced mechanical strength.

## 1. Introduction

Metal injection molding (MIM) is an emerging technology that combines traditional powder metallurgy, polymer chemistry, and plastic injection molding to accomplish the manufacturing of small and complex parts in bulk quantities [1,2,3,4]. In the MIM process, fine metal or ceramic powder is mixed with a measured amount of binder material to compose a feedstock, and this feedstock is then injected into a mold to produce the desired component. Following the molding process, the mechanical properties of the product are enhanced by performing a sintering process under carefully controlled temperature conditions [5]. The MIM process allows for the manufacture of complex parts in a single operation and with a high volume. The part surfaces have an exceptionally high flatness, equivalent to that generally achieved using a mechanical polishing process. Furthermore, due to the very small size of the powder particles (~10 micron), the precision and degree of freedom of the molding process are significantly improved, and the density and strength of the molded product following sintering are higher than those produced through conventional powder metallurgy [6]. As a result, MIM is widely applied nowadays; particularly for the manufacturing of products and components with high complexity, a small size and an exquisite appearance, such as medical supplies, smart phone hanging holes, and consumer electronics products. 

However, the MIM process still involves several critical challenges, including compensating for the volumetric shrinkage and warpage effects during the cooling stage of the molding process such that the final component satisfies the required geometric dimensions and tolerances, and improving the aesthetic appearance of the molded component by suppressing the formation of black lines on the product surface. Previous studies have shown that these black lines are caused by an excessive shear rate during the molding process, which prompts a phase separation of the powder particles and binding agent in the molding feedstock [7,8]. The black lines commonly occur in the vicinity of the injection gate and often require a follow-up process to facilitate their removal. Consequently, both the time and expense of the MIM process are inevitably increased.

In addition, since the weight of the metal powder injection molding product falls within the range of 0.030–300 g, and the procedure of degumming and sintering is still required, it is not easy to observe the metal powder distribution and defects from the surfaces of the initial injected parts, and the sintering process is required to be completed to obtain the information. To improve the mechanical and aesthetic quality of MIM products, it is essential that the effects of the main MIM processing parameters on the properties of the final product are properly understood [9,10]. However, due to the very small size of most MIM components, it is difficult to observe the metal powder distribution and surface defects of the injected part directly. Furthermore, the MIM processing conditions have both individual and interactive effects on the final molded part quality. Consequently, the use of experimental trial-and-error methods to determine the dominant factors which govern the final part quality is inefficient, time consuming and expensive. As a result, simulation methods are commonly preferred.

**Figure 1:**
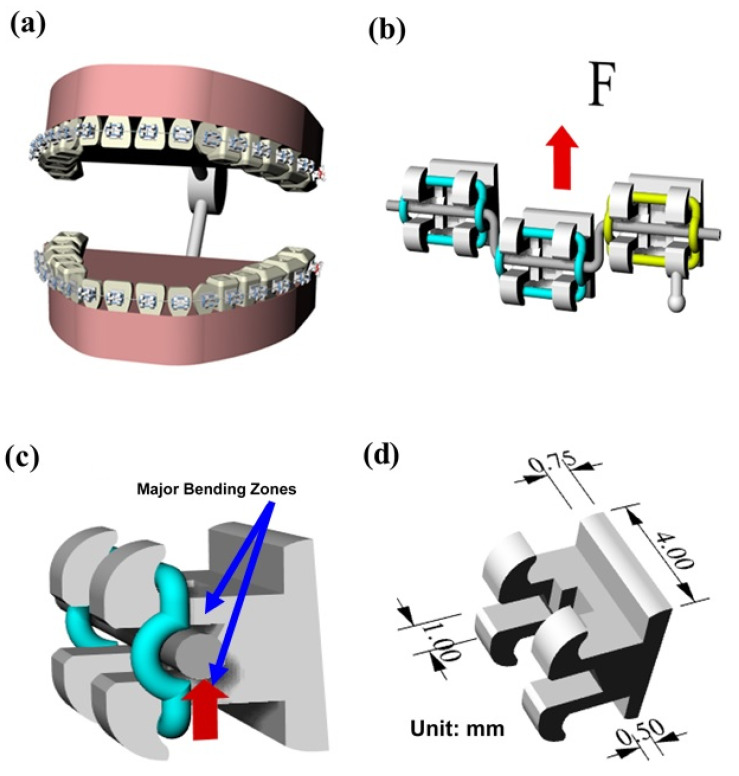
Orthodontic brace for oral correction: (**a**) general arrangement; (**b**) lateral forces acting on cantilever regions of orthodontic brace, (**c**) bending forces acting on cantilever regions of orthodontic brace, and (**d**) geometric dimensions of orthodontic brace.

The Taguchi method provides an approach for improving the quality of manufactured goods through a systematic process of statistical analysis and experimental trials [11]. Accordingly, the present study utilizes the Taguchi method to determine the optimal settings of the main MIM processing conditions (namely the filling time, the melt temperature, the packing pressure, the mold temperature, and the gate size) which improve the uniformity of the powder particle distribution during the MIM molding process and hence suppress the formation of black lines. The Taguchi method significantly reduces the number of experimental trials required to determine the optimal processing conditions compared to conventional trial-and-error methods. Nonetheless, the need for an experimental process still increases the time and expense of the optimization process. Therefore, the present study integrates the Taguchi design method with Moldex3D simulations [12,13] to determine the optimal MIM processing conditions in a more efficient and versatile manner. As shown in Figure 1, the study takes the case of an orthodontic brace as the research model. As shown in Figure 1b,c, the cantilever regions of the brace are subject to both lateral and bending forces under the effects of the arch wire. Consequently, the mechanical properties of the brace are of significant concern. Furthermore, the brace is easily visible to the casual observer when worn in the mouth. Thus, the aesthetic appearance of the brace (i.e., the absence of black lines on the surface) is also an important concern. As a result, the orthodontic brace represents an ideal target for the current integrated optimization approach. 

## 2. Effect and Black Line Formation

### 2.1. Shear-Induced Phase Separation Effect

**Figure 2:**
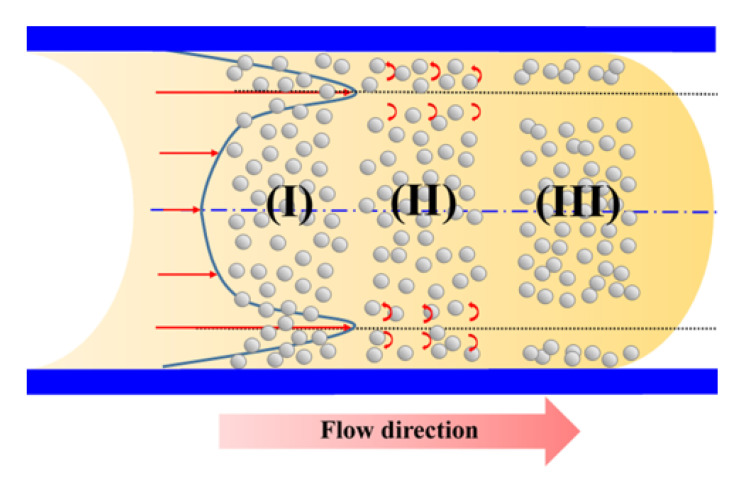
Shear-induced migration of metal powder particles in composite metal injection molding (MIM) material. Region (I): metal powder particles have random distribution within binder matrix; Region (II): metal powder particles rotate in different directions under effects of high shear rate gradient; Region (III): phase separation of metal powder particles and binder material.

In the powder injection molding process, a friction force is induced between the composite melt and the cavity wall as the melt flows through the gate and into the mold. As a result, a high shear rate gradient is induced within the channel, as shown in Figure 2. In the initial condition (Region I), the particles are randomly distributed throughout the binder material. However, the steep shear rate gradient induced by the friction force at either side of the channel causes the particles to undergo a local rotation effect, as shown in Region II. As the melt flow develops within the channel, the shear rate gradient causes a migration of the particles aligned with the points of maximum shear towards the center of the channel (see Region III). Hence, a phase separation of the metal powder particles and binder material occurs; resulting in a reduction in the powder concentration near the mold walls and an increase in the powder concentration in the center of the channel [14]. 

### 2.2. Formation of Black Lines

**Figure 3:**
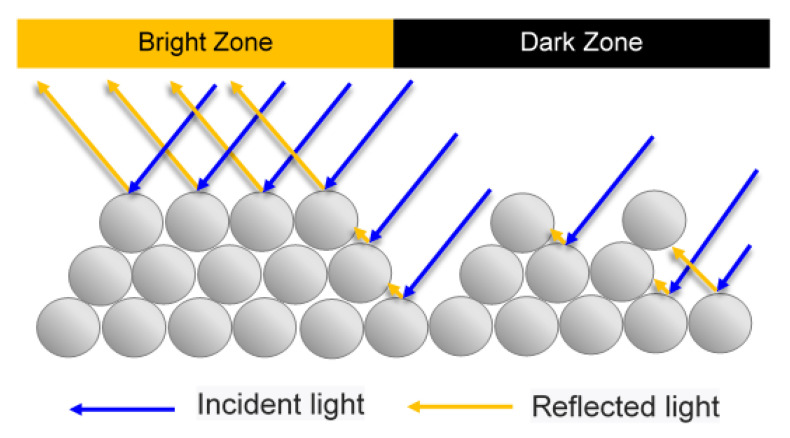
Schematic illustration showing formation of black lines under effects of different powder particle concentrations.

The difference in the powder concentration in the central and outer regions of the channel results in the formation of bright and dark zones on the surface of the molded product, as shown in Figure 3. In the regions of high powder concentration, the surface has a relatively high density and smoothness. Hence, the majority of the incident light is reflected directly, producing a bright and shiny surface. However, in the regions of low powder concentration, the surface is pitted and uneven. Consequently, some of the incident light is trapped by the powder particles, and hence the surface has a low and uneven gloss. As described above, the shaded regions of the sintered surface give rise to the formation of “black lines”, which severely degrade the aesthetic appearance of the molded product.

### 2.3. Metal Powder Concentration Distribution

To avoid the formation of the black lines described above, it is necessary to minimize the shear rate gradient of the melt flow within the mold during the molding process so as to improve the uniformity of the metal powder concentration distribution. In the present study, the uniformity of the powder concentration distribution under different MIM processing conditions is evaluated by computing the standard deviation of the powder particle concentration in accordance with Equation (1) below. In particular, the distribution of the metal powder concentration is divided into several intervals, where each interval is associated with a particular particle volume fraction, and Equation (1) is used to calculate the standard deviation between the actual powder concentration and the average powder concentration. A smaller value of the standard deviation indicates that the powder concentration has a smaller dispersion and is closer to the average value. In other words, a smaller standard deviation indicates a more uniform powder particle concentration distribution and hence, a reduced black line effect [15].

(1) $$\sigma \left(\mu \right)=\sqrt{\frac{1}{N}\overset{N}{\underset{i=1}{\sum }}{\left(x_{i}-\mu \right)}^{2}}$$

where *x_i_* is the value of each discrete sampling point, *μ* is the average value, *N* is the number of sampling points, and *i* is the sampling point index.

## 3. MIM Simulations

### 3.1. Mold Flow Analysis

**Figure 4:**
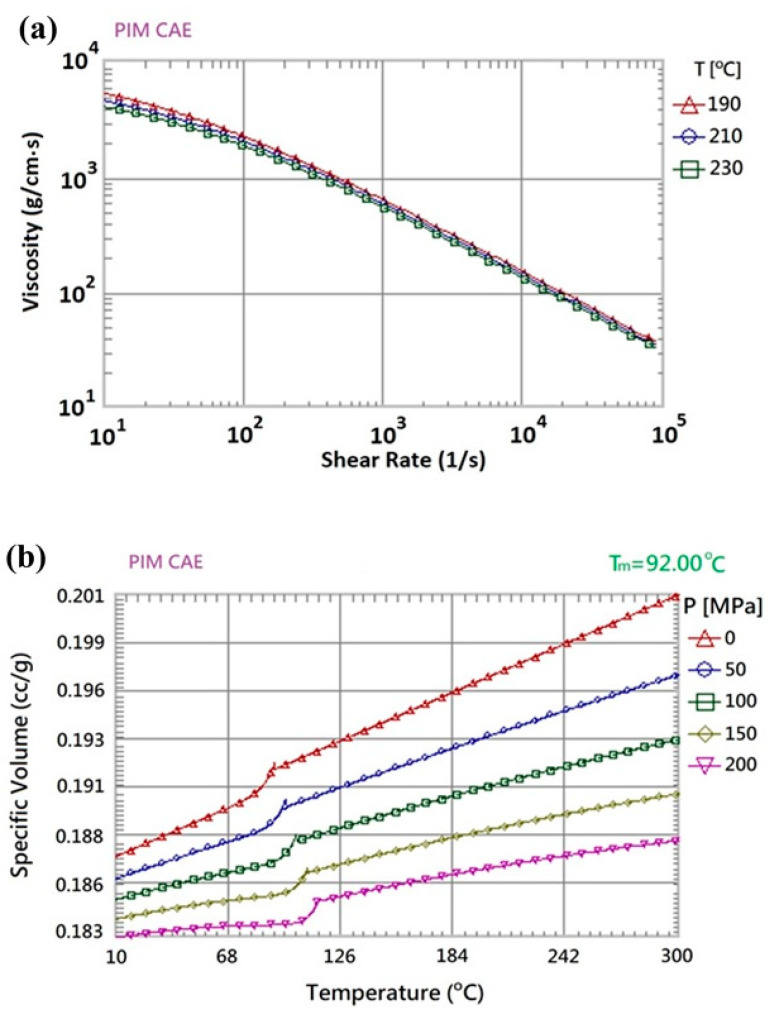
Material properties of composite material: (**a**) viscosity vs. shear rate and (**b**) specific volume vs. temperature.

In the present study, the powder particle concentration distribution within the molded component (i.e., the orthodontic brace) was evaluated by means of Moldex3D CAE simulations (CoreTech System Co., Ltd., Hsinchu, Taiwan). The brace had the dimensions shown in Figure 1d and was assumed to be fabricated using a composite material consisting of powder particles (stainless steel) with a volume fraction of 60% and a polymer binding agent (polypropylene, PP) with a volume fraction of 40%. The material information used by software developers—Moldex3D—is based on their laboratory using a specific manufacturing formula. The basic properties of the metal powder and binding agent are shown in Table 1, while the viscosity and specific volume properties are shown in Figure 4a,b, respectively. The mold flow analysis software treats the polymer fluid containing metal particles as a special pure fluid, and does not consider the variation of the nature and size of the internal particles.

The viscosity characteristics and specific volume properties of the material shown in Figure 4 are the formula parameters obtained by the software developer’s own laboratory for the measurement experiment of specially formulated polymer materials. The exact rheology relations are a database obtained under specific experimental conditions, and the other data obtained without experiments are subjected to the process of numerical interpolation/extrapolation under different conditions.

**Figure 5:**
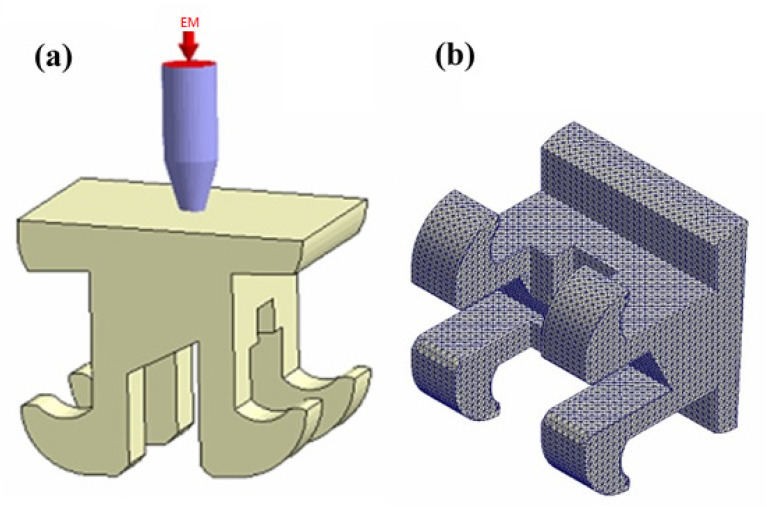
Simulation (**a**) model and (**b**) mesh of orthodontic brace.

As shown in Figure 5, the simulation model comprised an orthodontic brace and a single flow runner. In other words, the simulations considered a single-point infusion process (See Figure 5a). Boundary layer mesh (BLM) is an intuitive way to achieve high accuracy. BLM involves placing as many grid points (or meshes) as possible close to the mold wall. In this study, the phase separation effect occurs in the high shear rate layer adjacent to the mold wall. BLM grid technology can obtain more fluid information at the adjacent surface to accurately obtain the shear rate distribution and further evaluate the formation position of the black line [16,17]. Boundary layer mesh (BLM) technology is used to generate the 313,952 elements and 329,231 nodes in the orthodontic brace (See Figure 5b).

### 3.2. Taguchi Experiments

**Figure 6:**
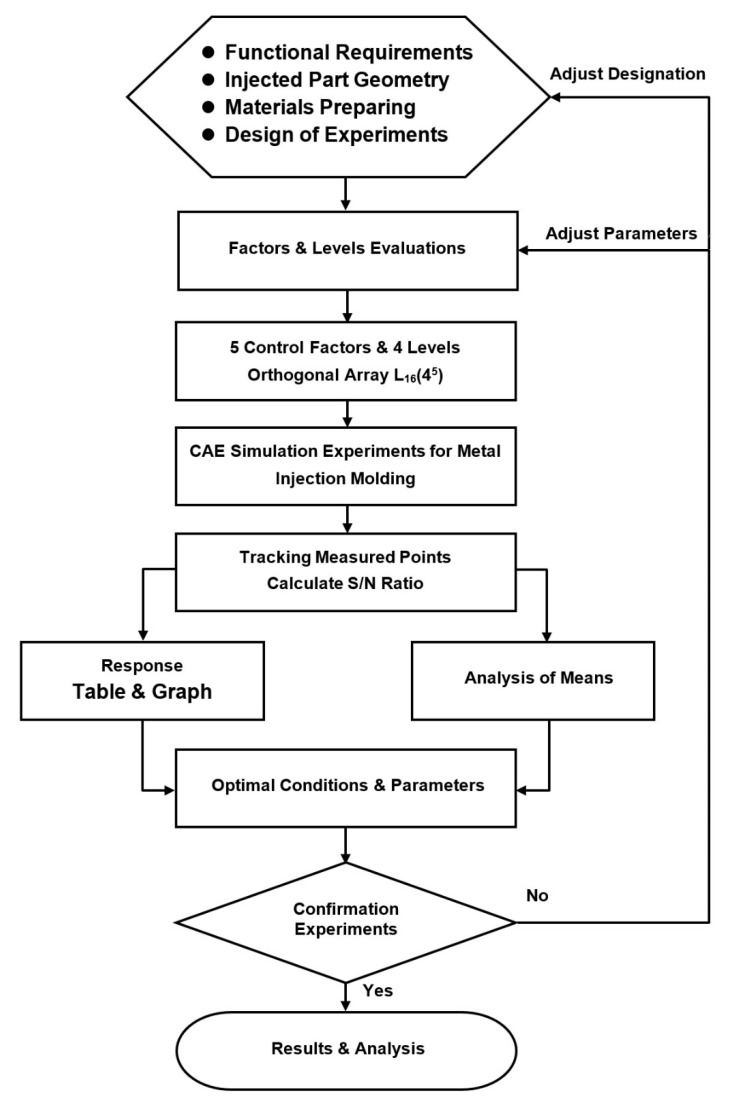
Flow chart of Taguchi-based MIM simulation framework. S/N: signal-to-noise.

The Taguchi method provides a powerful technique for determining the optimal processing conditions which render the output quality of manufacturing processes towards experimental variations. Importantly, the Taguchi method provides the maximum amount of information from a minimum number of experimental runs, and is hence extremely useful in optimizing complex processes with many inter-related variables, for which traditional trial-and-error approaches are inefficient, time consuming and expensive. In the present study, the Taguchi method was used to determine the optimal settings of the MIM processing conditions, which enhance the powder particle concentration uniformity within the molded brace and hence suppress the formation of black lines. Figure 6 presents a flow chart of the Taguchi/CAE simulation framework. In general, in implementing the Taguchi method, the quality of the outcome obtained from each experimental run is assessed using a signal-to-noise (S/N) approach [18]. Various forms of S/N ratio are available, depending on the particular problem under consideration. In the present study, the aim is to minimize the standard deviation metric shown in Equation (1), and hence the following smaller-the-better S/N ratio was employed:

(2) $$S/N=-10\log\left({\bar{y}}^{2}+S_{n}{}^{2}\right)$$

The simulations considered five control factors, namely (A) the filling time, (B) the packing pressure, (C) the melt temperature, (D) the mold temperature and (E) the gate size. Each factor was assigned four levels, as shown in Table 2. Orthogonal Array (OA) is a type of general fractional factorial design. It is based on an orthogonal design matrix, and allows the user to consider a selected subset of combinations of multiple factors at multiple levels. Orthogonal arrays are balanced to ensure that all levels of all factors are considered equally in statistics. Consequently, the experiments (simulations) were configured in an L_16_ (4^5^) orthogonal array with 16 trials, as shown in Table 3.

### 3.3. Factor Level Settings

**Figure 7:**
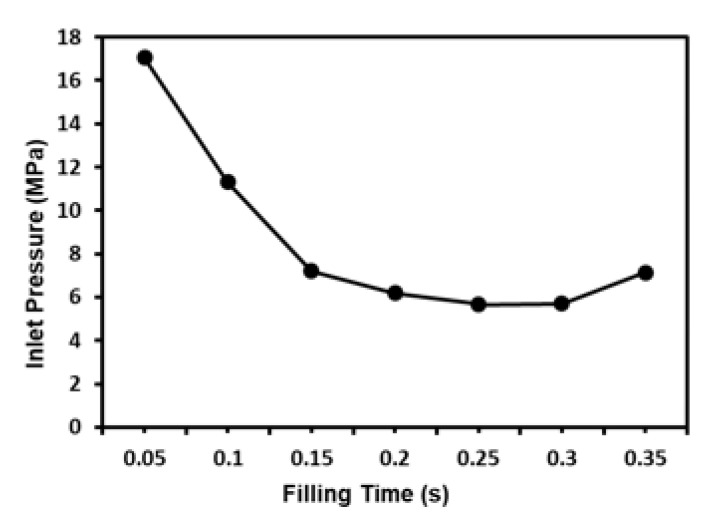
Correlation between inlet pressure and filling time.

**Figure 8:**
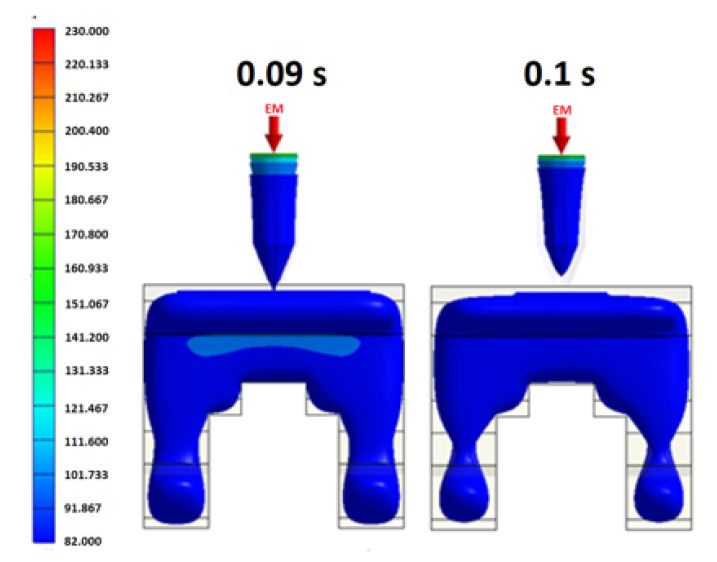
Temperature distribution in gate area of mold after packing times of 0.09 s (**left**) and 0.1 s (**right**).

For a given cavity volume, the filling time reduces as the injection pressure increases. However, an excessive injection pressure increases the flow stress near the gate and may therefore lead to product embrittlement. Furthermore, a higher injection pressure increases the residual stress within the product and causes an undesirable warpage and deformation behavior. Figure 7 shows the variation of the filling time with the inlet pressure for the present mold cavity (see Figure 5) based on the characteristics of a typical injection machine. As shown, the inlet pressure has a low and approximately constant value for filling times in the range of 0.15~0.3 s. Consequently, the factor level settings for the filling time were set as 0.15 s, 0.20 s, 0.25 s and 0.30 s, respectively (see Table 2). In the MIM process, the aim of the packing stage is to maximize the mold filling performance by applying an increased packing pressure in order to reduce the proportion of voids within the final product. To enhance the efficiency of the mold-filling process, the packing process must be performed while the melt in the gate region of the mold is still in a molten form. In other words, the duration of the packing process (i.e., the packing time) depends on the curing time at the gate. According to a series of preliminary simulations, the melt material in the gate region was completely solidified after a packing time of 0.1 s (see Figure 8). Given this packing time, the packing pressure was set in the range of 14~20 MPa in order to ensure an adequate filling of the mold and an acceptable molding quality. The feedstock in the MIM process has the form of a composite material consisting of a dispersion of metal powder particles within a binder matrix. For such materials, the viscosity reduces with an increasing melt and mold temperature (see Figure 4a) and hence leads to an improved uniformity of the metal powder particle distribution in the mold cavity. Based on the recommended processing temperature range for MIM compound materials specified in the Moldex3D-MIM software, the melt temperature level settings were set in the range of 190 °C to 230 °C and the mold temperature was set between 30 °C and 70 °C.

## 4. Results and Discussion

Mold flow simulations were performed for each experimental run in the L_16_ (4^5^) OA (Table 3). For each combination of the processing conditions, the average powder particle concentration and standard deviation of the powder particle concentration were computed directly from the simulation results. As described in Section 3.2, the aim of the simulation process was to determine the control factor level settings which produced the minimal standard deviation of the powder particle concentration within the brace, and hence minimized the appearance of black lines.

### 4.1. Relative Contributions of Control Factors

Table 3 shows the mean and standard deviation values of the powder particle concentration for each of the 16 experimental runs in the Taguchi OA. Table 4 shows the S/N response of the four levels of each of the five control factors. An inspection of Table 4 shows that the filling time (Factor A), with a standard deviation range of 0.211784 dB and a 41.9% contribution, has the greatest effect on the metal powder distribution in the molded brace. The melt temperature (Factor C), with a standard deviation range of 0.176641 dB and a 34.96% contribution, also has a large effect on the uniformity of the powder particle concentration distribution. By contrast, the remaining factors (i.e., the packing pressure, the mold temperature and the gate size) all have only a limited effect on the particle distribution (i.e., 11.64%, 7.3% and 4.2% contributions, respectively). 

The finding that the metal powder concentration is dominated by the filling time and melt temperature is reasonable since both factors have a significant effect on the melt viscosity. In particular, a shorter filling time and higher melt rate are both associated with a reduced melt viscosity and therefore a lower shear rate during filling. Consequently, the phase separation effect is reduced, and the uniformity of the particle distribution within the molded component is improved. As shown in Table 4, the gate size has the least effect of the considered factors on the powder particle concentration. In general, a smaller gate size increases the overall metal powder concentration and enhances the strength of the molded part. However, if the gate diameter is too small, it impedes the flow of the melt into the mold and prompts a separation of the metal powder particles and binding agent.

### 4.2. Optimal Factor Level Settings

**Figure 9:**
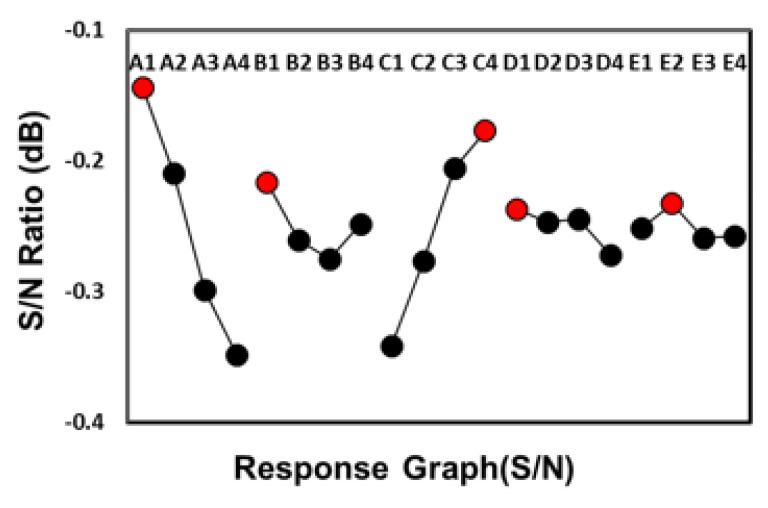
S/N response graph for Taguchi experiments.

Figure 9 shows the S/N factor response graph for the 16 experimental runs in the Taguchi OA. For each control factor, the level setting which yields the maximum S/N ratio value (i.e., the maximum particle concentration uniformity) is indicated in red. The results show that the optimal processing conditions are as follows: Factor A: filling time 0.15 s, Factor B: packing pressure 14 MPa, Factor C: melt temperature 230 °C, Factor D: mold temperature 30 °C, and Factor E: gate size 0.25 mm.

### 4.3. Verification of Optimal Processing Parameters

**Figure 10:**
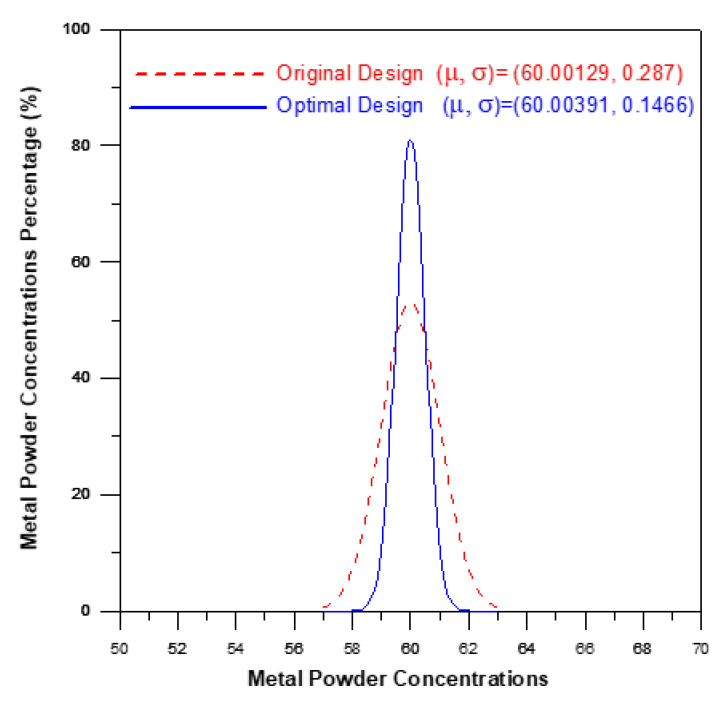
Comparison of metal powder concentration distributions in original process design and optimal process design.

**Figure 11:**
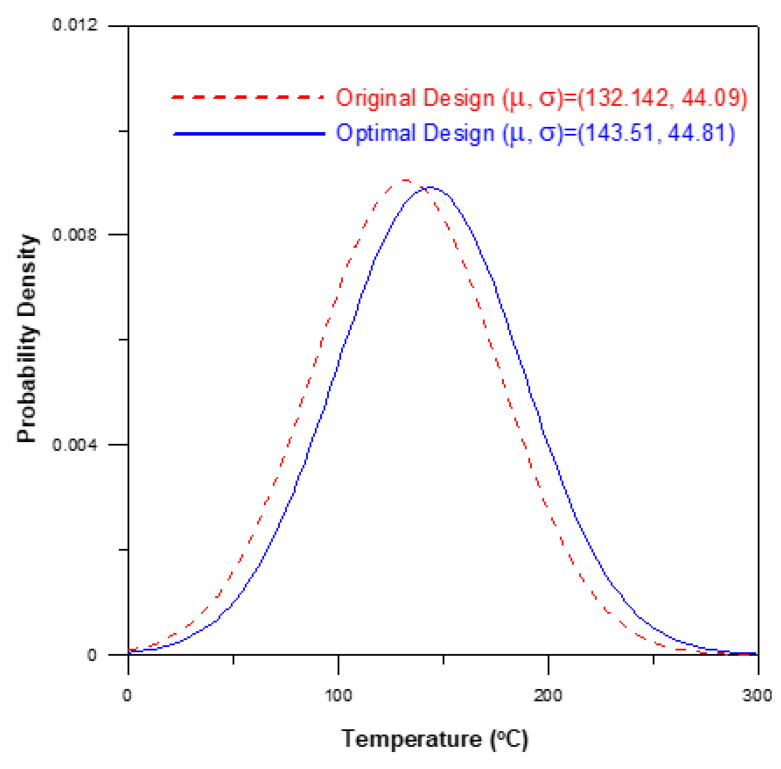
Comparison of temperature distributions in original process design and optimal process design.

A verification experiment was performed to confirm the effectiveness of the optimal process parameter settings described above in improving the uniformity of the powder particle distribution within the brace. For comparison purposes, the optimized results obtained for the powder particle concentration distribution were compared with those obtained using the processing conditions shown in the 12th run of the Taguchi OA. Figure 10 compares the simulation results obtained for the powder particle concentration distribution given the use of the original processing parameters and the optimal processing parameters, respectively. The results confirm that the optimal processing parameters yield a more uniform concentration of the powder particles. In particular, the optimal parameters reduce the standard deviation of the powder particle concentration from 0.287 in the original process design (corresponding to a S/N ratio of −0.3455 dB (see Table 3)) to 0.1466 (corresponding to a S/N ratio of −0.1191 dB). Figure 11 shows the average and standard deviation values of the temperature in the mold under the original and optimal process designs, respectively. It is seen that the optimal process parameters increase the average temperature by around 11 °C (i.e., 143.51 °C–132.142 °C). As a result, the flow viscosity of the melt reduces and prompts a corresponding reduction in the shear effect. Consequently, the phase separation effect is also diminished, and hence the uniformity of the powder particle concentration distribution is improved.

### 4.4. Relationship between Metal Powder Concentration Distribution and Black Line Formation

**Figure 12:**
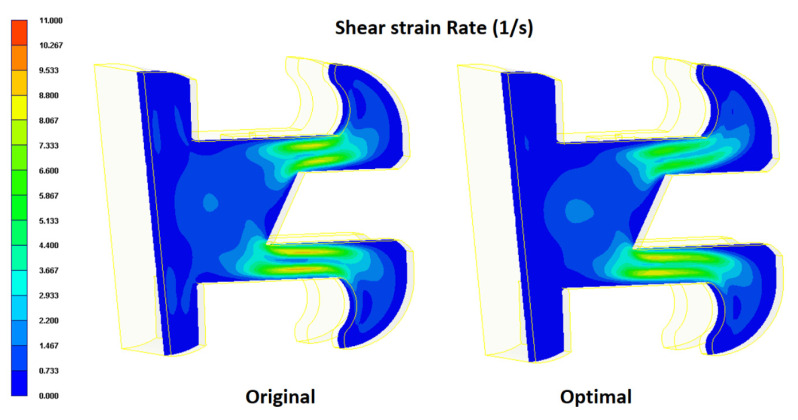
Comparison of shear strain rate distributions in original process design and optimal process design.

**Figure 13:**
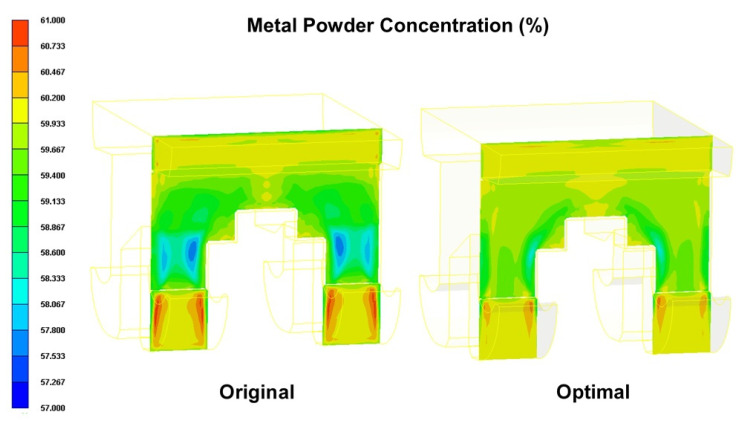
Comparison of metal powder concentration distributions in original process design and optimal process design.

**Figure 14:**
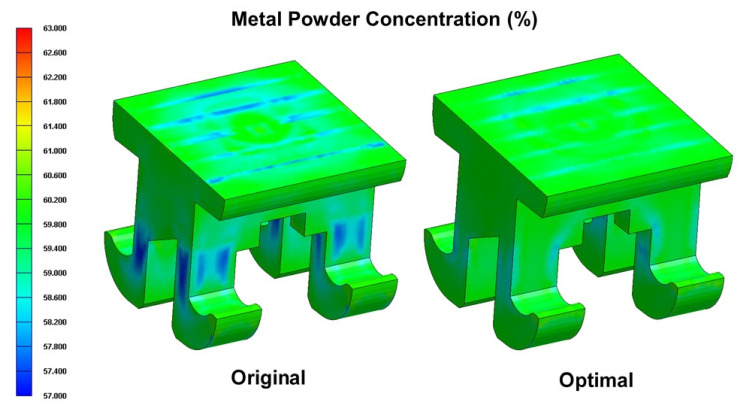
Comparison of metal powder concentration distributions on surface of orthodontic brace in original process design and optimal process design.

The cantilever arms of the orthodontic brace experience a significant bending moment under the forcing effects of the arch wire (see Figure 1). Consequently, the mechanical properties of the brace in the cantilever arm regions are of particular concern. As shown in Figure 12, the shear strain rate in the cantilever arms of the orthodontic brace is significantly higher than that in the other regions of the brace due to the reduced cross-sectional area of the cavity. However, comparing the two figures for the original process design and the optimal process design, respectively, it can be seen that the optimal parameter settings yield a smaller value of the shear strain rate. In other words, it can be inferred that the optimal process parameters reduce the phase separation effect in the cantilever regions of the brace. Figure 13 and Figure 14 present cross-sectional and surface views of the powder particle distribution within the brace, given the use of the original process design and the optimal process design, respectively. As discussed previously in Figure 10, the optimized process conditions reduce the standard deviation of the powder particle concentration distribution from 0.297 to 0.1466; corresponding to an improvement of 48.92%. The simulation results presented in Figure 13 and Figure 14 confirm that the optimal process parameters yield a more uniform distribution of the powder particle concentration in both the cross-sectional and surface planes of the brace, and hence inhibit the formation of black lines.

## 5. Conclusions

This study has employed the Taguchi design methodology and mold flow analysis simulations to investigate the optimal MIM processing conditions which minimize the formation of black lines on the surface of the molded product (an orthodontic brace). In performing the simulations, it has been assumed that the formation of the black lines is directly related to the concentration distribution of the metal particles within the mold. It has been further assumed that the concentration distribution of the particles is directly related to the magnitude of the shear strain rate induced in the filling process. In other words, the aim of the Taguchi experiments is to determine the optimal MIM processing conditions which minimize the shear strain rate and hence, improve the uniformity of the powder particle concentration distribution. The simulation results support the following main conclusions.

1. The MIM control factors can be ranked in terms of a decreasing effect on the powder particle concentration distribution as follows: filling time > melt temperature > packing pressure > mold temperature > gate size.
2. The uniformity of the powder particle concentration distribution can be improved by using a shorter filling time, a higher melt temperature, a lower packing pressure, a lower mold temperature, and a smaller gate size.
3. For the orthodontic brace considered in the present study (see Figure 1), the optimal processing conditions are as follows: (A) filling time 0.15 s, (B) packing pressure 14 MPa, (C) melt temperature 230 °C, (D) mold temperature 30 °C, and (E) gate size 0.25 mm.
4. The optimal processing conditions reduce the standard deviation of the particle powder concentration distribution by 48.92% compared to that in the original process design.
5. The optimal processing conditions yield an effective reduction in the peak shear strain rate induced during the molding process. Consequently, the phase separation effect of the metal powder particles within the composite feedstock material is also reduced, and hence the formation of black lines on the surface of the molded brace is suppressed.

## Figures and Tables

**Table 1:** Metal powder and binding agent used in MIM simulations.

	Metal Powder	Binding Agent
**Specifics**	Stainless steel	Polypropylene (PP)
**Volume fraction**	60%	40%
**Density**	7.9 g/cc	0.91 g/cc
**Particle dimension**	10 microns	-

**Table 2:** Control factors and level settings used in Taguchi experiments.

L_16_ (4^5^)	A	B	C	D	E
	Filling Time (s)	Packing Pressure (MPa)	Melt Temperature ( ℃ )	Mold Temperature ( ℃ )	Gate Size (mm)
**Level 1**	0.15	14	200	30	0.2
**Level 2**	0.2	16	210	40	0.25
**Level 3**	0.25	18	220	50	0.3
**Level 4**	0.3	20	230	60	0.35

**Table 3:** Taguchi L_16_ (4^5^) orthogonal array.

Exp.	A Filling Time (s)	B Packing Pressure (MPa)	C Melt Temperature ( ℃ )	D Mold Temperature ( ℃ )	E Gate Size (mm)	$\bar{y}$ (60%)	S/N (dB)
1	0.15	14	200	30	0.2	1.00103	−0.2179
2	0.15	16	210	40	0.25	1.00065	−0.1902
3	0.15	18	220	50	0.3	1.0003	−0.1509
4	0.15	20	230	60	0.35	1.00015	−0.1194
5	0.2	14	210	50	0.35	1.00015	−0.2329
6	0.2	16	200	60	0.3	1.00023	−0.3732
7	0.2	18	230	30	0.25	1.0006	−0.1547
8	0.2	20	220	40	0.2	1.00103	−0.1799
9	0.25	14	220	60	0.25	1.00056	−0.2588
10	0.25	16	230	50	0.2	1.00103	−0.2548
11	0.25	18	200	40	0.35	1.00009	−0.4556
12	0.25	20	210	30	0.3	1.00022	−0.3455
13	0.3	14	230	40	0.3	1.0002	−0.2709
14	0.3	16	220	30	0.35	1.0001	−0.3404
15	0.3	18	210	60	0.2	1.00106	−0.4546
16	0.3	20	200	50	0.25	1.00051	−0.4597
Optimization	0.15	14	230	30	0.25	1.00065	−0.1191

**Table 4:** S/N response of control factors and level settings in Taguchi experiments.

S/N(dB)	A	B	C	D	E
**Level 1**	−0.16961	−0.24516	−0.37662	−0.26464	−0.27684
**Level 2**	−0.23521	−0.28966	−0.30581	−0.27416	−0.26584
**Level 3**	−0.32868	−0.30395	−0.23249	−0.27458	−0.28511
**Level 4**	−0.38139	−0.27612	−0.19998	−0.30151	−0.28709
**Range**	0.211784	0.058797	0.176641	0.036867	0.021249
**Contribution**	41.90%	11.64%	34.96%	7.30%	4.20%
**Rank**	1	3	2	4	5
